# Inference of brain pathway activities for Alzheimer's disease classification

**DOI:** 10.1186/1472-6947-15-S1-S1

**Published:** 2015-05-20

**Authors:** Jongan Lee, Younghoon Kim, Yong Jeong, Duk L Na, Jong-Won Kim, Kwang H Lee, Doheon Lee

**Affiliations:** 1 Department of Laboratory Medicine and Genetics, Samsung Medical Center, Sungkyunkwan University School of Medicine, Seoul, South Korea; 2 Department of Bio and Brain Engineering, KAIST, Daejeon, South Korea; 3 Department of Neurology, Samsung Medical Center, Sungkyunkwan University School of Medicine, Seoul, South Korea

## Abstract

**Background:**

Alzheimer's disease (AD) is a neurodegenerative and progressive disorder that results in brain malfunctions. Resting-state (RS) functional magnetic resonance imaging (fMRI) techniques have been successfully applied for quantifying brain activities of both Alzheimer's disease (AD) and amnestic mild cognitive impairment (aMCI) patients. Region-based approaches are widely utilized to classify patients from cognitively normal subjects (CN). Nevertheless, region-based approaches have a few limitations, reproducibility owing to selection of disease-specific brain regions, and heterogeneity of brain activities during disease progression. For coping with these issues, network-based approaches have been suggested in the field of molecular bioinformatics. In comparison with individual gene-based approaches, they acquired more accurate results in diverse disease classification, and reproducibility was confirmed by replication studies. In our work, we applied a similar methodology integrating brain pathway information into pathway activity inference, and permitting classification of both aMCI and AD patients based on pathway activities rather than single region activities.

**Results:**

After aggregating the 59 brain pathways from literature, we estimated brain pathway activities by using exhaustive search algorithms between patients and cognitively normal subjects, and identified discriminatory pathways according to disease progression. We used three different data sets and each data set consists of two different groups. Our results show that the pathway-based approach (AUC = 0.89, 0.9, 0.75) outperformed the region-based approach (AUC = 0.69, 0.8, 0.68). Also, our approach provided enhanced diagnostic power achieving higher accuracy, sensitivity, and specificity (pathway-based approach: accuracy = 83%; sensitivity = 86%; specificity = 78%, region-based approach: accuracy = 74%; sensitivity = 78%; specificity = 76%).

**Conclusions:**

We proposed a novel method inferring brain pathway activities for disease classification. Our approach shows better classification performance than region-based approach in four classification models. We expect that brain pathway-based approach would be helpful for precise classification of brain disorders, and provide new opportunities for uncovering disrupted brain pathways caused by disease. Moreover, discriminatory pathways between patients and cognitively normal subjects may facilitate the interpretation of functional alterations during disease progression.

## Background

Resting-state (RS) functional magnetic resonance imaging (fMRI) technology has been used to quantify functional brain connectivity and neuronal changes of Alzheimer's disease (AD) [1]. In the resting condition, functional alterations of brain disorders are measured by spontaneous neuronal activity derived from blood-oxygen-level-dependent (BOLD) signal fluctuations even in the absence of external stimulus. Abnormalities in functional communication of brain disorders are useful indicators for classification between patients and CN subjects.

A few RS-fMRI methods have been suggested for estimating the functional alterations between AD patients and CN subjects [2]. Among the model-dependent approaches, region-based approaches are typically used to investigate the local functional connectivity changes of AD patients, because they are sensitive for detecting abnormalities of functional connections within disease-specific regions, and interpretation of disease symptoms is simple [3-5]. Region-based approaches estimate correlations of BOLD signals between pre-defined regions of interest (ROIs). However, region-based approaches have a few limitations to discriminate both AD and aMCI patients from CN subjects, because classification analysis using limited brain regions has difficulties for evaluating functional alterations in the whole brains. These approaches are dependent on pre-defined brain regions, and thus pathological mechanisms during AD progression could not be explained in the whole brain. Region-based approaches focus on functional connectivity within disease-specific regions, such as the hippocampus or the posterior cingulate cortex (PCC) [6,7]. Also, well-defined disease information is necessary to determine candidate brain regions considering structural and functional system. To overcome drawbacks of region-based approaches, a few studies implemented large-scale RS-fMRI analysis for investigating functional connectivity changes in the whole brain [8,9]. The whole brain regions were divided into hierarchically structured regions including frontal lobe, temporal lobe, parietal lobe, occipital lobe, and limbic system. The large-scale approaches can globally detect the functional connectivity changes between paired brain regions in the whole brain. However, significantly diverse patterns of cognitive and functional decline in individual AD patients were reported by using the quantitative measurements [10]. These diverse patterns caused by cognitive decline and functional degeneration lead to different functional connectivities in AD patients. Hence, the heterogeneous connectivity patterns during AD progression have to be seriously considered for precise disease classification.

Here, we propose a novel brain pathway-based classification method to address these challenges. Our approach incorporates brain pathway information into inferring pathway activities, and then pathway activities were estimated between patients with AD, aMCI and CN subjects. The 59 brain pathways information was collected from the literature, and these pathways contain brain connectivity, lateralization, and associated functions. These brain pathways were selected to cover behavioral domains such as cognition, perception, sensation, motor, and emotion function. Brain pathways consist of functionally associated brain regions which are validated by biological experiments or brain imaging studies. In cognitively normal subject, functionally specialized regions are connected each other to process information in brain pathways, while their functional connectivities might be disrupted in both aMCI and AD patients. Interestingly, network-based approaches in the field of molecular bioinformatics have been successfully applied to cope with heterogeneity problems of samples [11]. They have achieved more accurate and reproducible classification, and discovered interpretable markers by incorporating molecular pathway information into disease classification [12,13]. Similarly, we evaluated pathway activities inferred from brain pathways rather than single region activities for discriminating patients with aMCI, AD from CN subjects. In resting-state brain, the spontaneous neural activations of cognitively normal subjects were identified in behavioral domains such as vision, audition, sensory-motor system [14,15]. We hypothesis that spontaneous neural activities between aMCI and AD patients are abnormal versus CN subjects in behavioral domains, and these phenomena result in disrupted brain activities at rest. In comparison with region-based approaches, the brain pathway-based approach could overcome heterogeneity problems through pathway activity inference. Also, our approach could provide new opportunities for unveiling dysregulated brain pathways during AD progression.

## Methods

### Subjects

This study was approved by the Institutional Review Board of Samsung Medical Center. Written informed consent was obtained from aMCI, AD patients, and cognitively normal (CN) subjects. One hundred twenty right-handed subjects were recruited through the Samsung Medical Center: 22 cognitively normal (CN) subjects, 37 aMCI patients, 61 AD patients (21 patients with very mild AD, 27 patients with mild AD, 13 patients with moderate AD). The AD stages were categorized by the National Institute of Neurological and Communicative Disorders and Stroke/ Alzheimer's Disease and Related Disorders Association (NINCDS-ADRDA) criteria [16]. For the diagnosis of aMCI patients, Mayo Clinic criteria were used [17].

### fMRI imaging

Magnetic resonance imaging (MRI) examination was conducted on a 3.0-T MR scanner 3.0 T scanner (Model; Philips Intera Achieva, Phillips Healthcare, Netherlands). Scans involved the acquisition of 35 axial slices using a gradient echo planar imaging pulse sequence: repetition time (TR) = 3000 ms; echo time (TE) = 35 ms; acquisition time (TA) = 5 minutes; flip angle (FA) = 90°; field of view (FOV) (RL, AP, FH) = 220 × 220 × 140 mm; voxel size (RL, AP) = 2.875 mm × 2.875 mm with a slice thickness of 4 mm. During the scan, participants were instructed to lie still with their eyes open. Additionally T1-weighted anatomical images were obtained for each subject: TR = 1114 ms; TE = 10 ms; FA = 8°; FOV (RL, AP, FH) = 220 × 220 × 132 mm; REC voxel size = 0.43 mm × 0.43 mm × 0.43 mm.

Preprocessing of MR image data was performed by the FMRIB Software Library, FSL 4.1 [18]. The first 6 volumes from the functional MRI runs were discarded to avoid T1 equilibrium effects. Then, the following pre-statistic processing steps were done: deleting non-brain tissues from images using a Brain Extraction Tool (BET), motion correction using MCFLIRT [19]. Grand mean intensity normalization of the whole 4D data set by a single multiplicative factor, spatial smoothing using a Gaussian kernel of FWHM 5 mm, high pass temporal filtering (Gaussian-weighted least-squares straight line fitting, with sigma = 50 to ensure at least half power was preserved for frequencies down to 0.01 Hz). The corrected MR images were registered into the Montreal Neurological Institute space (MNI-152 stereotactic template) using FLIRT, FMRIB's linear image registration tool.

### Integration of brain pathways

Brain pathways are comprised of anatomically separated regions, but functionally connected regions. Brain pathways were characterized by biological functions and behavioral domains such as perception, motor, cognition, emotion, and sensation. These brain pathways have been discovered and revised by in vivo and in vitro experiments. For example, the Papez pathway was regarded as the emotional pathway, but it was revised as the limbic system pathway through other experimental validations [20].

In our study, the 59 brain pathways were selected based on the behavioral domains, the associated functions, and lateralization (Table 1). Brain pathways were divided into the left (L) and the right (R) brain hemisphere except for 7 pathways. The 7 pathways are dominantly lateralized in the left or the right brain hemisphere. In the default mode network (DMN), the positively correlated networks of both ventromedial prefrontal cortex (vmPFC) and posterior cingulate cortex (PCC) regions were used as the brain pathways. Among 59 brain pathways, 38 pathways are well-known brain pathways covering systemic neuroscience [21,22]. Other 21 pathways were manually curated from literature to supplement 38 well-known pathways, considering specialized functional and structural systems of the brain in cognitively normal subjects [23-39]. For example, emotional domains of 38 pathways describe general features of the emotion, however manually curated Krolak-Salmon (2004) pathway explain more detailed phenomena of fear spreading in emotional domain. These 21 pathways were aggregated with in vitro imaging studies, such as diffusion tensor imaging (DTI), electroencephalography (EEG), structural MRI, and functional MRI. The manually curated 21 brain pathways were named according to last name of first authors and publication years. The regional connectivity and lateralization of 59 brain pathways are described in Additional file 1.

**Table 1:** The 59 Brain pathways with behavioral domains, associated functions, and lateralization.

Behavioral domain	Associated functions	Brain pathways	Lateralization	Reference
Cognition	Executive function	Dorsolateral prefrontal	left,right	[23]
	Decision making	Orbitofrontal	left,right	[23]
	Attention	Medial prefrontal	left,right	[23]
	Motivation	Anterior cingulate	left,right	[24]
	Memory storage	Papez	left,right	[24]
	Repeat spoken word	Language(auditory)	left	[22]
	Repeat written word	Language(visual)	left	[22]
	Fairness decisions	Baumgartner (2011)	right	[25]
	Efficient reading	Richardson (2011)	left,right	[26]
	Language	Frey (2008)	left	[27]
	Memory	Ji (2007)	left,right	[28]
	Decision making	Walton (2004)	left,right	[29]
	Error observation	Van Schie (2004)	left,right	[30]
	Reading	Turkeltaub (2003)	left	[31]
	Spontaneous thought	DMN	left,right	[32]
	Learning	Benchenane (2010)	left,right	[33]

Emotion	Fear conditioning	Emotion(fear)	left,right	[22]
	Emotion processing	Emotion	left,right	[21]
	Facial expression	Emotion(expression)	left,right	[21]
	Renewal of fear	Orsini (2011)	left,right	[34]
	Fear spreading	Krolak-Salmon (2004)	left	[35]

Motor	Control of movement	Motor	left,right	[36]
	Limb movement	Cerebellar	left,right	[22]

Sensation	Vision sensation	Visual	left,right	[22]
	Hearing sensation	Auditory	left,right	[22]
	Taste sensation	Gustatory	left,right	[22]
	Touch, pain	Somatosensory	left,right	[22]
	Smell sensation	Olfactory	left,right	[22]
	Aversive taste	Nitschke (2006)	left,right	[37]
	Attention to odor	Plailly (2008)	left,right	[38]

Recognition	Spatial vision	Visual(dorsal)	left,right	[22]
	Object recognition	Visual(ventral)	left,right	[22]
	Face recognition	Druzgal (2001)	left	[39]

### Functional connectivity

The whole brain was divided into 116 brain regions based on a gray matter mask with atlas labels by using the automated anatomical labeling (AAL) atlas [40]. A hierarchical segmentation of the whole brain covers the cortical regions (frontal cortex, temporal cortex, parietal cortex, and occipital cortex), subcortical regions (limbic regions, insula, basal ganglia, thalamus), and cerebellar hemisphere (Additional file 2). Using gray matter masks, the averaged MR signals of 116 brain regions of both CN subjects and patients were extracted by FSL tool.

**Figure 1:**
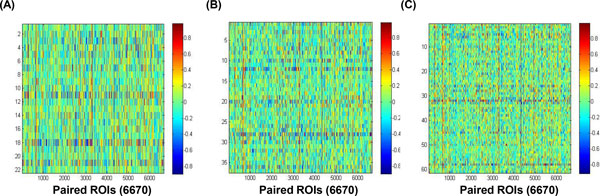
**Functional connectivity (R) matrix between paired ROIs**. (A) CN subjects (B) aMCI patients (C) AD patients. Each matrix has the 6670 *r *values calculated from combination of 116 brain regions. The color bar indicates the Pearson's correlation coefficient (*r*) values.

The functional connectivities between paired brain regions were measured by the Pearson's correlation coefficient (*r*). The types of functional connectivity were categorized according to strengths of linear relationships. Positive correlations between paired regions indicate that MR signals of one brain region were increased, and the other brain region has a tendency to also increase, while negative correlations has a tendency to decrease. Also, no correlations between paired regions show that the other brain region does not tend to either decrease or increase. As a result, the 6670 (116 × 115 / 2) *r *values between paired brain regions were produced of both CN subjects and patients, and these *r *values were arranged into the functional connectivity (R) matrix (Figure 1). Strengths of functional connectivities between paired regions were represented by color bars: red color indicates positive correlations; blue color indicates negative correlations; green color indicates no correlations.

### Inferring pathway activity for the brain pathway-based approach

**Figure 2:**
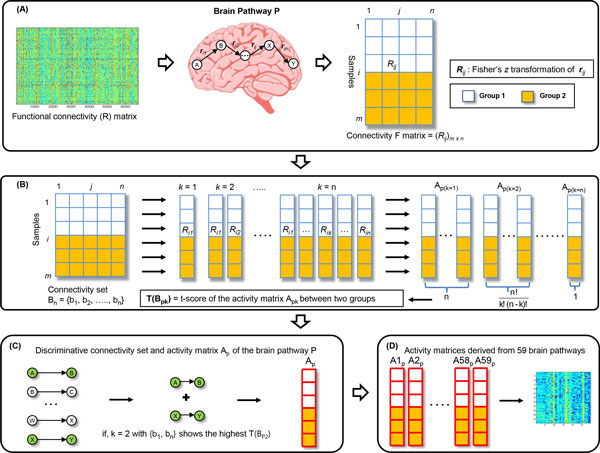
**Schematic diagram of the brain pathway activity inference**. For the given brain pathway P, the activity A_p _matrix was acquired through the exhaustive search between two different groups.

In this work, we use three different data sets described in Method, and each data set consists of two different groups: the first data set (between 22 CN subjects and 37 aMCI patients); the second data set (between 22 CN subjects and 61 AD patients); the third data set (between 37 aMCI and 61 AD patients). Pearson's correlation coefficient (*r*) values of all sample *i *over functional connectivity *j *were arranged into the connectivity (F) matrix to aggregate the functional connectivities between paired brain regions corresponding the brain pathway P (Figure 2A). For example, the orbitofrontal pathway has sequential connections from orbitofrontal cortex to caudate, globus pallidus, thalamus, and orbitofrontal cortex [23]. The *r *values of each data set (*r*_1 _between orbitofrontal cortex and caudate, *r*_2 _between caudate and globus pallidus, *r*_3 _between globus pallidus and thalamus, *r*_4 _between thalamus and orbitofrontal cortex) were arranged in the connectivity (F) matrix. Fisher's *z *transformation was applied to *r *values to obtain the normal distributed values *R_ij _*of all samples *i *over functional connectivity *j*. As a result, the connectivity (F) matrix was acquired between two groups in each data set.

The exhaustive search was performed to identify the discriminative connectivity set in connectivity (F) matrix (Figure 2B). To detect dominant signals between two groups, all the possible connectivities in connectivity (F) matrix were considered through the exhaustive search, and optimally discriminative connectivity set between two groups was selected according to their statistical significance. All the possible combinations of the functional connectivities in the connectivity set B_n _were considered from *k *= 1 to *k *= n. All *R_ij _*values in the connectivity set B_k _was transformed into averaged *R *values which were designated the activity score a_pk_.

$$\text{Activity}\left({\text{a}}_{\text{pk}}\right)\text{score}=\overset{k}{\underset{s=1}{\sum }}\frac{R_{is}}{k}$$

All the possible combinations in the connectivity set B_n, _and activity matrices A_pk_

$$k=1\to \left\{{\text{b}}_{1}\right\},\left\{{\text{b}}_{2}\right\},\ldots \;\;\ldots ,\left\{{\text{b}}_{\text{n}}\right\}\to \text{A}1_{\text{p}1},\text{A}2_{\text{p1}},\ldots \;\ldots ,\text{A}{\text{n}}_{\text{p}1}$$

$$k=2\to \left\{{\text{b}}_{1},{\text{b}}_{2}\right\},\left\{{\text{b}}_{1},{\text{b}}_{3}\right\},\ldots ,\left\{{\text{b}}_{\text{n - 1}},{\text{b}}_{\text{n}}\right\}\to \text{A}1_{\text{p}2},\text{A}2_{\text{p2}},\ldots \;,\text{A}{\frac{\text{n!}}{\text{k!(n}-\text{k)!}}}_{\text{p}2}$$

……

$$k=\text{n}\to \left\{{\text{b}}_{1},{\text{b}}_{2}\text{,}\ldots \;\;\ldots \text{,}{\text{b}}_{\text{n}}\right\}\to \text{A}1_{\text{pn}}$$

We defined the t-score T(B_pk_) of the activity matrices A_pk _derived from connectivity set B_k _from *k *= 1 to *k *= n. The normality of the activity matrices A_pk _was confirmed by Kolmogorov-Smirnov tests (p-value > 0.05, normal distribution), and the equality of variance was assessed by Levene's tests (p-value < 0.05, unequal variance) for the 2-tail t-test. In the brain pathway P, the discriminative connectivity set was defined when the T(B_pk_) score reaches much higher than other t-score values among the activity matrices A_pk_. The higher T(B_pk_) score between two groups indicates statistical differences, and their connectivity set was regarded as quantitative activity indices of brain pathways. The activity matrix A_p _acquired from the discriminative connectivity set between two groups was assigned as the brain pathway P activity. If *k *= 2 with connectivity set {b_1_, b_n_} shows the highest T(B_p2_) score among all the possible combinations of connectivity set B_n_, and the activity matrix A_p _of connectivity set {b_1_, b_n_} was designated as the brain pathway P activity (Figure 2C).

**Figure 3:**
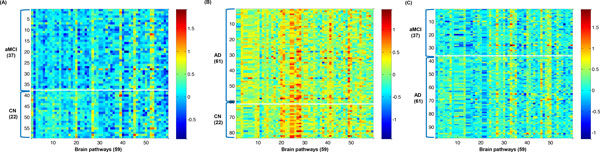
**Inferred activity matrices using 59 brain pathways between two different groups**. (A) between CN subjects and aMCI patients (B) between CN subjects and AD patients (C) between aMCI and AD patients.

The discriminative connectivity set between two groups indicates brain malfunctions corresponding specific brain pathway. For example, if functions of memory in the brain are disrupted during AD progression, functional connectivities of memory pathways in AD patients show abnormal patterns versus cognitively normal subjects. These unusual patterns of functional connectivities are selected as the discriminative connectivity set between cognitively normal subjects and AD patients. As a result, we obtained the 59 activity matrices between two groups across 59 brain pathways (Figure 2D). In three different data sets, the 59 activity matrices were generated between two groups. To clearly differentiate 59 brain activities between two groups, they were rearranged according to statistical significance (Figure 3).

### Regional functional correlation strength for the region-based approach

The activities of 59 brain pathways were used as input features for the brain pathway-based classification. In order to compare the brain pathway-based approach with the region-based approach in an unbiased manner, we used the same number of input features for the region-based approach. For selecting 59 input features of the region-based approach, we estimated regional functional correlation strength (RFCS) using previously described method in imaging study [41]. In region a, the correlation strength was defined as:

$$\text{Correlation}\;\text{Strengt}{\text{h}}_{\text{region}}\left(a\right)=\frac{1}{N-1}\underset{i\neq j}{\sum }|r_{ab}|$$

where *r_ab _*is the Pearson's correlation coefficient (*r*) values between brain region a and b, and N is the number of brain regions.

**Figure 4:**
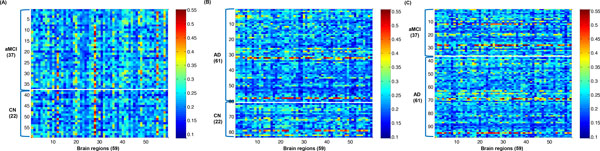
**The 59 RFCS matrices between two different groups among 116 brain regions**. (A) between CN subjects and aMCI patients (B) between CN subjects and AD patients (C) between aMCI and AD patients.

From the functional connectivity (R) matrix, we acquired the RFCS scores of 116 regions between two different groups, and then 2-tail t-test was performed between two different groups to select the RFCS scores of 59 brain regions as input features for the seed-based approach. The RFCS scores of 116 brain regions were rearranged by their t-test score in ascending order, and then the RFSC scores of top 59 brain regions were selected as input features for the seed-based approach. As a result, we acquired the 59 RFCS matrices between two groups across 59 brain regions in three different data sets (Figure 4).

### Evaluation methods for classification

We used the four supervised machine learning algorithms for evaluating the classification performance. In three different data sets, we trained on both 59 activity matrices (pathway-based approach) and 59 RFSC matrices (region-based approach) by using three linear classifiers: Naïve Bayes (NB); logistic regression; support vector machine (SVM) and one decision trees classifier: random forest (RF).

All samples in both 59 activity matrices and 59 RFSC matrices were randomly partitioned into ten equivalent subsamples. Among the ten subsamples, nine subsamples were used as training data set for building classifiers, and one subsample was remained for testing the classification models. The process of cross-validation was repeated 10 times, and each of ten subsamples employed just once as the test set (10-fold cross-validation). Each fold calculated classification accuracy, and results of ten folds were averaged to create a single evaluation. Performance of four classifiers was estimated by the area under the curve (AUC) in the receiver operating characteristics (ROC), accuracy, sensitivity and specificity.

$$\text{Accuracy}=\frac{\left(\text{TP}+\text{TN}\right)}{\left(\text{TP}+\text{FP}+\text{TN}+\text{FN}\right)}$$

$$\text{Sensitivity}=\frac{\text{TP}}{\left(\text{TP}+\text{FN}\right)}$$

$$\text{Specificity}=\frac{\text{TN}}{\left(\text{FP}+\text{TN}\right)}$$

TP: True Positive, FP: False Positive, TN: True Negative, FN: False Negative

### Feature selection

The quantification of the brain pathways importance is necessary for interpretation of pathological symptoms during AD progression. For identifying discriminatory pathways between two groups, the feature selection was performed by using the random forest (RF). RF is the efficient algorithm for solving classification problems, because classification performance of RF model is enhanced by growing an ensemble of trees and letting them vote for the most preferable group [42].

There are two scoring methods for measuring variable importance with RF: mean decrease accuracy (MDA), mean decrease Gini (MDG). Between two scoring methods, we adopted the MDA for evaluating variable importance within two groups; variables having higher MDA values contribute importantly toward the classification, and variables having lower MDA values could not affect the classification. After calculating MDA scores between two groups, we ranked the variables (59 brain pathways) according to MDA scores, and selected the top-K variables as significant features. The K values were determined by the distribution of MDA scores, and we defined the decreasing points of MDA scores when slopes of distributions is dramatically changed, and selected the variables before the first changing point in the distribution as important features.

## Results

### Comparison of classification performance

Unlike NB and logistic regression classifier, classification performance of both RF and SVM models were affected by setting parameters. Among four kernel functions of SVM model, we used the RBF kernel with c and gamma parameters. The gamma parameter is the radius of RBF kernel, and the c parameter controls the importance of the training error with respect to the margin. Large c values provide us low bias and high variance, while small c values give us high bias and low variance. The SVM classification model were estimated by tuning gamma and c parameters, and their AUC values were reported. The values of gamma and c parameters were used in the range between 0.0001 and 10000. In three different data sets, the AUC values of RF classification model were measured by adjusting the number of trees from 100 to 1000, and then 500 trees were fixed.

**Figure 5:**
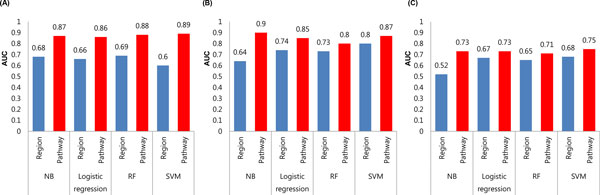
**Performance comparison of two different approaches in three different data sets**. Bar chart of the area under ROC curve (AUC): blue color (region-based approach), red color (pathway-based approach). (A) between CN subjects and aMCI patients (B) between CN subjects and AD patients (C) between aMCI and AD patients.

The classification performance of both the brain pathway-based approach and the region-based approach was shown by evaluating the area under the ROC curve (AUC) with the four different classification models (Figure 5). Overall, the brain pathway-based approach outperformed the region-based approach in three different data sets. In the brain pathway-based approach, the best performance was yielded by the SVM classification model except for between AD patients and CN subjects (between aMCI patients and CN subjects, 0.89; between AD patients and CN subjects, 0.87; between aMCI and AD patients, 0.75). The highest AUC values of the SVM model in the brain pathway-based approach were achieved by different combination of gamma and c parameters: between aMCI patients and CN subjects (c, 0.0001; gamma, 0.0001), between AD patients and CN subjects (c, 1000; gamma, 0.01), between aMCI and AD patients (c, 0.0001; gamma, 0.001). Between AD patients and CN subjects, the highest classification performance in brain pathway-based approach was achieved by the NB classification model (AUC = 0.9).

Similarly, the region-based approach yielded the best performance in the SVM classification model except for between aMCI patients and CN subjects (between aMCI patients and CN subjects, 0.6; between AD patients and CN subjects, 0.8; between aMCI and AD patients, 0.68). Between aMCI patients and CN subjects, the highest classification performance in region-based approach was achieved by the RF classification model (AUC = 0.69). The highest AUC values of the SVM model in region-based approach were acquired by combination of both the c and gamma values: between aMCI patients and CN subjects (c, 0.001; gamma, 10), between AD patients and CN subjects (c, 100; gamma, 0.1), between aMCI and AD patients (c, 10000; gamma, 0.0001).

### Comparison of diagnostic power

Table 2 shows that, for classification accuracy, sensitivity, and specificity of two groups in three different data sets. In comparison with the region-based approach, the pathway-based approach consistently achieved more accurate classification between aMCI, AD patients and CN subjects. For classifying aMCI patients from CN subjects, the SVM classification model of the pathway-based approach achieved the optimal diagnostic power (accuracy, 83%; sensitivity, 86%; specificity, 78%), while the region-based approach achieved the best diagnostic power (accuracy, 62%; sensitivity, 76%; specificity, 51%). In case of AD classification, the logistic regression model of the pathway-based approach showed the optimal diagnostic power (accuracy, 83%; sensitivity, 86%; specificity, 83%).

**Table 2:** Comparison of diagnostic power between brain pathway-based approach and region-based approach.

(A) Pathway-based approach									
**Classification Models**	**aMCI vs. CN**			**AD vs. CN**			**aMCI vs. AD**		
	
	**Acc(%)**	**Sen(%)**	**Spe(%)**	**Acc(%)**	**Sen(%)**	**Spe(%)**	**Acc(%)**	**Sen(%)**	**Spe(%)**

NB	77	83	73	81	86	78	68	70	63
Logistic regression	72	81	69	83	86	83	66	72	64
RF	77	78	67	79	83	73	69	70	61
SVM	83	86	78	79	85	73	67	70	64

**(B) Region-based approach**									

**Classification**	**aMCI vs. CN**			**AD vs. CN**			**aMCI vs. AD**		
	
**Models**	**Acc(%)**	**Sen(%)**	**Spe(%)**	**Acc(%)**	**Sen(%)**	**Spe(%)**	**Acc(%)**	**Sen(%)**	**Spe(%)**

NB	61	67	60	73	70	50	57	56	53
Logistic regression	59	59	55	72	68	64	62	70	60
RF	61	64	69	74	72	64	60	78	61
SVM	62	76	51	74	78	69	66	72	60

Acc = Accuracy, Sen = Sensitivity, Spe = Specificity

Similarly, for classifying aMCI from AD patients, the pathway-based approach outperformed the region-based approach in four different classification models. Table 2 also indicates that, there are slight differences of both accuracy and sensitivity of each classification model, while the differences of specificity are relatively large in the pathway-based approach. Both aMCI and AD classification with CN subjects, these characteristics of high sensitivity might be beneficial for considering diagnostic aspects, because the cost for misclassifying as patients into CN subjects is much higher than vice versa.

### Identification of discriminatory pathways

To select discriminatory brain pathways among 59 brain pathways, MDA values were calculated by the RF classification model. The discriminatory brain pathways were acquired by variable importance graph described in Method. The default mode network (PCC) was consistently selected by important features to discriminate two groups in three different data sets (Table 3).

**Table 3:** Discriminatory brain pathways between two groups of three different data sets.

Rank	aMCI vs. CN	AD vs. CN	aMCI vs. AD
1	Right Nitschke (2006)	Left Emotion fear	Left Motor
2	Left Turkeltaub (2003)	Default mode network (PCC)	Right Dorsal visual
3	Right Orbitofrontal	Right Papez	Right Nitschke (2006)
4	Right Motor	Default mode network (vmPFC)	Default mode network (PCC)
5	Default mode network (PCC)	Left Papez	Right Papez
6		Left Somatosensory	

Between aMCI patients and CN subjects, three well-known and two manually curated brain pathways were chosen as the discriminatory pathways. The selected five brain pathways cover diverse behavioral domains; cognition, sensation and motor functions. Between AD patients and CN subjects, the six well-known brain pathways were acquired as the discriminatory pathways. Compared with the first data set between aMCI patients and CN subjects, the emotional domain (top rank) related to fear conditioning was additionally selected. Between aMCI and AD patients, the four well-known and one manually curated brain pathways were chosen as the discriminatory pathways. Compared with the first data set between aMCI patients and CN subjects, the recognition domain (second rank) was also selected.

## Discussion

Although the RS-fMRI technique using region-based approaches has become an effective tool to discriminate patients from CN subjects, several issues still remained. Region-based approaches require the prior knowledge of brain regions related to the brain disorders. On the other hand, the brain pathway-based approach is unrestricted by these problems. Our approach could elucidate pathological phenomena in the course of AD progression, and it yields robust classification performance regardless of the classification models. Here, we explain pathological symptoms in both aMCI and AD patients by comparing our results with those of previous studies.

Between aMCI patients and CN subjects, the right Nitschke (2006) pathway was selected as the most important feature, and this pathway is related to the aversive taste in the sensory domain. From the previous study, the quantitative and qualitative taste functions of patients with 29 MCI and 30 AD patients were investigated by the taste strips test [43]. The total taste scores of both MCI and AD patients were considerably decreased as compared with healthy subjects. Second, the left Turkeltaub (2003) pathway is related to reading in the cognitive domain. In prior experiments, the significant reduction of word-specific activation in 13 aMCI patients was reported by chronometric analysis of word reading and picture naming [44]. Third, the structural and functional alterations of the default mode network (DMN) in AD patients were reported by using imaging techniques such as RS-fMRI, positron emission tomography (PET) [45,46]. Recently, the decreased and dysfunctional DMN connectivity in MCI patients was detected by comparison with healthy subjects [47,48].

Between AD patients and CN subjects, the left emotional pathway was considered as the most significant feature. Deficits of emotional processing were reported in both visual and auditory domain [49]. In both aMCI and AD patients, the default mode network (DMN) was obtained as important variables. Those results could elucidate progressive and degenerative phenomena during AD progression. Other selected discriminatory pathways are related to the function of sensation and memory. First, the left somatosensory pathway has an important role to produce sensory modalities such as touch, pain, and body position. For testing the somatosensory response in both MCI and AD, MEG responses were measured and analyzed with semi-automated source localization algorithm [50]. They found that the primary somatosensory cortex was affected in the early AD patients. Second, the Papez pathway has the functions in memory storage and emotion control. The integrity of Papez circuit (hippocampus, fornix, mammillary bodies, thalamus, cingulate cortex) in AD patients was investigated in vivo and at post-mortem [51]. Between aMCI and AD patients, the left motor pathway was chosen as the most significant variable. One study reported a relationship between upper and lower extremity motor function and functional impairment by testing 371 probable AD patients [52].

Heterogeneity in the course of AD was investigated using quantitative measurement, the Global Deterioration Scale (GDS) and Functional Assessment Staging procedure (FAST) [10]. Significantly diverse patterns of cognitive and functional decline were found in corresponding individual AD patients. Considering the heterogeneity in the course of AD, the region-based approaches have weak points since these approaches are dependent on the functional connectivity of AD between paired regions. In the course of AD progress, the diversity of functional connectivity raises several problems for the precise AD classification. Thus, in response to the heterogeneity of AD patients, more comprehensive approaches are necessary rather than limited region-based approaches. Interestingly, our results showed homogenous aspects in discriminatory brain pathways as an important variable to differentiate both aMCI and AD patients from CN subjects. In both aMCI and AD patients, the discriminatory pathways concerning cognitive functions were concurrently selected by the important feature. From these results, we could effectively detect functional alterations using brain pathway information regardless of the AD stages. Also, the brain pathway-based approach does not depend on any assumption or hypothesis, such as the hippocampal connectivity or small world network. We can observe global dysfunction of the brain in the progression of AD. We identified functionally disrupted pathways between CN subjects and patients in the cognition, motor, emotion, sensation, and recognition domain. Our approach provides effective interpretation using discriminatory brain pathways between CN subjects and patients. Moreover, our method yielded better classification performance compared to the seed-based approach regardless of both classification models and AD stages.

## Conclusions

To examine the alterations in brain activities among anatomical brain regions, we used pathway-based approach in both cognitively normal (CN) subjects and Alzheimer's disease (AD), amnestic mild cognitive impairment (aMCI) patients. We suggested the new method incorporating brain pathway information into discriminatory analysis between two groups, and inferred brain activities by using 59 brain pathways show better classification performance than the region-based approach in four classification models. Also, our approach provided significantly increased diagnostic power achieving higher accuracy, sensitivity, and specificity in between aMCI, AD patients and CN subjects.

The major contribution of this work is twofold. First, we could cope with individual heterogeneity problems owing to disease progression by using pathway information, because brain regions in specific pathway connect with each other to process the information. Second, discriminatory pathways between groups could provide neurologist with a clue to explain pathological symptoms and investigate potential candidates in the course of AD. Additionally, brain connectivity databases are increasing gradually at present, allowing further opportunities to discover novel brain pathways along AD stages.

## Competing interests

The authors declare that they have no competing interests.

## Authors' contributions

JL designed the method, validated results and wrote the manuscript, YK performed experiments and wrote the manuscript. YJ and DLN collected experimental data. JYK participated in experiment design. KHL and DL supervised the study and revised the manuscripts. All authors reviewed and approved the manuscript.

## Supplementary Material

Additional file 1 **The regional connectivity and lateralization of 59 brain pathways**. Click here for file

Additional file 2 **Automatically parcellated 116 brain regions**. Click here for file

## Declarations

The publication costs for this article were funded by the corresponding author.

This article has been published as part of *BMC Medical Informatics and Decision Making *Volume 15 Supplement 1, 2015: Proceedings of the ACM Eighth International Workshop on Data and Text Mining in Biomedical Informatics. The full contents of the supplement are available online at http://www.biomedcentral.com/bmcmedinformdecismak/supplements/15/S1.
