# Supplementary material for: Inference of brain pathway activities for Alzheimer's disease classification
Source: BMC Med Inform Decis Mak. 2015 May 20;15(Suppl 1):S1. doi: 10.1186/1472-6947-15-S1-S1 (PMC4460780; doi:10.1186/1472-6947-15-S1-S1)
Supplement: Additional file 1 — The regional connectivity and lateralization of 59 brain pathways. [file 1472-6947-15-S1-S1-S1.pdf]

Additional file 1 The regional connectivity and lateralization of 59 brain pathways.

Brain pathway	Lateralization	Regional Connectivity
Dorsolateral prefrontal	left,right	Dorsolateral prefrontal cortex → Caudate → Globus pallidus → Thalamus → Dorsolateral prefrontal cortex
Orbitofrontal	left,right	Orbitofrontal cortex → Caudate → Globus pallidus → Thalamus →Orbitofrontal cortex
Medial prefrontal	left,right	Medial prefrontal cortex → Nucleus accumbens → Globus pallidus → Thalamus → Medial prefrontal cortex
Anterior cingulate	left,right	Anterior cingulate cortex → Striatum → Globus pallidus → Thalamus → Anterior cingulate gyrus
Papez	left,right	Anterior cingulate cortex → Hippocampus → Mamillary body → Thalamus → Anterior cingulate cortex
Language(auditory)	left	Primary auditory cortex → Angular gyrus → Wernicke's area → Broca's area → SMA → Primary motor cortex
Language(visual)	left	Primary visual cortex → Angular gyrus → Wernicke's area → Broca's area → SMA → Primary motor cortex
Baumgartner (2011)	right	Right dorsolateral prefrontal cortex → right posterior ventromedial prefrontal cortex
Richardson (2011)	left,right	posterior inferior occipital → ventral occipitotemporal area → posterior superior temporal sulcus → anterior superior temporal sulcus
Frey (2008)	left	Inferior frontal gyrus pars opercularis → Inferior parietal lobule
		inferior frontal gyrus pars triangularis → Superior temporal gyrus, Heschl's gyrus
Ji (2007)	left,right	Visual cortex → Hippocampus
Walton (2004)	left,right	Orbitofrontal cortex → Anterior cingulate cortex
Van Schie (2004)	left,right	Medial frontal cortex → Motor cortex
Turkeltaub (2003)	left	left middle temporal → left inferior frontal gyrus
Default mode network(vmPFC)	left,right	Medial frontal gyrus(R) → Superior frontal gyrus(L), Inferior frontal gyrus(R), Inferior frontal gyrus(L), Precuneus(R), Middle temporal gyrus(R), Angular Gyrus(R), Cingulate Gyrus(R), Middle temporal gyrus(L), Middle temporal gyrus(R), Parahippocampal gyrus(R), Middle temporal gyrus(L), Parahippocampal gyrus (L), Middle frontal gyrus(R), Middle frontal gyrus(L)
Default mode network(PCC)	left,right	Posterior cingulate gyrus(R) → Angular gyrus(R), Medial frontal gyrus(R) Angular gyrus(L), Medial frontal gyrus(L), Inferior temporal gyrus(R), Middle frontal gyrus(R), Inferior temporal gyrus(L), Superior frontal gyrus(R), Superior temporal gyrus(R), Superior frontal gyrus(L), Fusiform gyrus(R), Superior frontal gyrus(R), Inferior frontal gyrus(L), Postcentral gyrus(R) Middle frontal gyrus(L),Middle frontal gyrus(R), Middle frontal gyrus(L)
Benchenane (2010)	left,right	hippocampus → medial prefrontal cortex
Emotion(fear)	left,right	Thalamus → Primary sensory cortex → Hippocampus → amygdala
Emotion	left,right	Thalamus → Primary visual cortex → Amygdala → Anterior cingulate gyrus, ventromedial prefrontal cortex
Emotion(expression)	left,right	Globus pallidus → Putamen → Caudate
Orsini (2011)	left,right	Hippocampus → Amygdala, Medial prefrontal cortex → Amygdala
Krolak-Salmon (2004)	left	Amygdala → Occipitotemporal cortex, Anterior temporal cortex, Orbitofrontal cortex
Motor	left,right	Supplementary motor area (SMA) → Putamen → Globus pallidus → Thalamus → SMA
Cerebellar	left,right	Cerebellum → thalamus → Primary motor cortex → SMA
Visual	left,right	Thalamus → Primary Visual cortex (calcarine sulcus)
Auditory	left,right	Thalamus → Primary Auditory cortex (heschl’s gyrus)
Gustatory	left,right	Thalamus → Primary Gustatory cortex (insula, parietal operculum)
Somatosensory	left,right	Thalamus → Primary Somatosensory cortex (postcentral gyrus)
Olfactory	left,right	Thalamus → Primary Olfactory cortex
Nitschke (2006)	left,right	Insula → Frontal operculum, Parietal operculum
Plailly (2008)	left,right	Olfactory cortex → Thalamus → Orbirofrontal cortex
Visual(dorsal)	left,right	Primary visual cortex → Posterior parietal cortex (superior parietal lobule)
Visual(ventral)	left,right	Primary visual cortex → Inferior temporal cortex
Druzgal (2001)	left	Anterior cingulate cortex → Left fusiform face area, Left dorsolateral prefrontal cortex
