# Supplementary material for: Inference of brain pathway activities for Alzheimer's disease classification
Source: BMC Med Inform Decis Mak. 2015 May 20;15(Suppl 1):S1. doi: 10.1186/1472-6947-15-S1-S1 (PMC4460780; doi:10.1186/1472-6947-15-S1-S1)
Supplement: Additional file 2 — Automatically parcellated 116 brain regions. [file 1472-6947-15-S1-S1-S2.pdf]

Additional file 2 Automatically parcellated 116 brain regions.

	Left hemisphere	Right hemisphere
Central region	Left Precentral gyrus	Right Precentral gyrus
	Left Postcentral gyrus	Right Postcentral gyrus
	Left Rolandic operculum	Right Rolandic operculum
Frontal lobe	Left Superior frontal gyrus, dorsolateral	Right Superior frontal gyrus, dorsolateral
	Left Middle frontal gyrus	Right Middle frontal gyrus
	Left Inferior frontal gyrus, opercular	Right Inferior frontal gyrus, opercular
	Left Inferior frontal gyrus, triangular	Right Inferior frontal gyrus, triangular
	Left Superior frontal gyrus, medial	Right Superior frontal gyrus, medial
	Left Supplementary motor	Right Supplementary motor
	Left Paracentral lobule	Right Paracentral lobule
	Left Superior frontal gyrus, orbital	Right Superior frontal gyrus, orbital
	Left Superior frontal gyrus, medial orbital	Right Superior frontal gyrus, medial orbital
	Left Middle frontal gyrus, orbital part	Right Middle frontal gyrus, orbital part
	Left Inferior frontal gyrus, orbital part	Right Inferior frontal gyrus, orbital part
	Left Gyrus rectus	Right Gyrus rectus
	Left Olfactory cortex	Right Olfactory cortex
Temporal lobe	Left Superior temporal gyrus	Right Superior temporal gyrus
	Left Heschl gyrus	Right Heschl gyrus
	Left Middle temporal gyrus	Right Middle temporal gyrus
	Left Inferior temporal gyrus	Right Inferior temporal gyrus
Parietal lobe	Left Superior parietal gyrus	Right Superior parietal gyrus
	Left Inferior parietal	Right Inferior parietal
	Left Angular gyrus	Right Angular gyrus
	Left Supramarginal gyrus	Right Supramarginal gyrus
	Left Precuneus	Right Precuneus
Occipital lobe	Left Superior occipital gyrus	Right Superior occipital gyrus
	Left Middle occipital gyrus	Right Middle occipital gyrus
	Left Inferior occipital gyrus	Right Inferior occipital gyrus
	Left Cuneus	Right Cuneus
	Left Calcarine sulcus	Right Calcarine sulcus
	Left Lingual gyrus	Right Lingual gyrus
	Left Fusiform gyrus	Right Fusiform gyrus
Limbic lobe	Left Superior temporal pole	Right Superior temporal pole
	Left Middle temporal pole	Right Middle temporal pole
	Left Anterior cingulate gyrus	Right Anterior cingulate gyrus
	Left Middle cingulate gyrus	Right Middle cingulate gyrus
	Left Posterior cingulate gyrus	Right Posterior cingulate gyrus
	Left Hippocampus	Right Hippocampus
	Left Parahippocampal gyrus	Right Parahippocampal gyrus
Subcortical gray nuclei	Left Amygdala	Right Amygdala
	Left Caudate	Right Caudate
	Left Putamen	Right Putamen
	Left Pallidum	Right Pallidum
	Left Thalamus	Right Thalamus
	Left Insula	Right Insula
Cerebellum	Left Cerebellum Crus1	Right Cerebellum Crus1
	Left Cerebellum Crus2	Right Cerebellum Crus2
	Left Cerebellum 3	Right Cerebellum 3
	Left Cerebellum 4, 5	Right Cerebellum 4, 5
	Left Cerebellum 6	Right Cerebellum 6
	Left Cerebellum 7B	Right Cerebellum 7B
	Left Cerebellum 8	Right Cerebellum 8
	Left Cerebellum 9	Right Cerebellum 9
	Left Cerebellum 10	Right Cerebellum 10
	Left Vermis 1,2	Right Vermis 1,2
	Left Vermis 3	Right Vermis 3
	Left Vermis 4, 5	Right Vermis 4, 5
	Left Vermis 6	Right Vermis 6
	Left Vermis 7	Right Vermis 7
	Left Vermis 8	Right Vermis 8
	Left Vermis 9	Right Vermis 9
